# Passive fit of milled and additively manufactured complete arch implant-supported titanium frameworks: a micro-CT analysis

**DOI:** 10.1186/s12903-026-07702-2

**Published:** 2026-02-10

**Authors:** Heba Shawky Eid, Mohamed Radwan, Liam Blunt, Paul Bills, Ahmed Tawfik, Mazen A. Attia

**Affiliations:** 1 Department of Fixed Prosthodontics, Faculty of Dentistry, Ain Shams University, Cairo, Egypt; 2 Department of Fixed Prosthodontics, Faculty of Dentistry, Beni-Suef University, Beni-Suef, Egypt; 3 EPSRC Future Advanced Metrology Hub, University of Huddersfield, Huddersfield, United Kingdom

## Abstract

**Background:**

To evaluate the passive fit of milled and additively manufactured titanium complete arch implant-supported frameworks (CAISFs) by using microcomputed tomography (µCT).

**Methods:**

A maxillary model resembling all-on-4 concept was used, multiunit abutments and scan bodies were attached, and the model was digitized. Ten frameworks were produced from CAD files and allocated to 2 groups (*n=*5) according to the manufacturing method: milled titanium (M) and additively manufactured titanium (AM). The passive fit of CAISFs was evaluated by using the modified 1-screw test. All screws were attached and then removed, except for the terminal abutment tightened to 15 Ncm. The Primary outcome to access passive fit involved measuring marginal discrepancies for the 3 unscrewed implants at 4 points using µCT. Mann-Whitney U test assessed manufacturing methods. Kruskal-Wallis test assessed effect of implant locations and different measuring surfaces on marginal discrepancy. Aligned rank transform (ART) ANOVA was used to evaluate the effect of the interactions among all factors (α=.05).

**Results:**

Regarding the passive fit, significant difference in marginal discrepancy value was found between the 2 manufacturing methods (*P*<.001). AM group had lower marginal discrepancy value (44 ±4 μm) than M group (49 ±5 μm). No significant differences in marginal discrepancy value were found among the 3 implant locations (*P*=.822). Statistically significant differences were found among the 4 measurement surfaces (mesial, buccal, lingual, distal) (*P*<.001). ART ANOVA showed that the interaction among manufacturing method, implant location, and measurement surface was not statistically significant (*P*=.992).

**Conclusion:**

Additively manufactured titanium CAISFs demonstrated better passive fit than milled ones. However, both additive manufacturing and milling techniques produced titanium CAISFs with clinically acceptable marginal discrepancy. The proximity of the implant location to the screwed implant did not affect the marginal discrepancy.

**Clinical implications:**

Additively manufactured and milled titanium frameworks demonstrated superior marginal fit, suggesting their potential applicability and advantage in clinical practice.

## Background

Because of their biocompatibility, corrosion resistance, and superior mechanical properties, titanium and its alloys have been used in prosthetic dentistry to fabricate complete arch implant-supported frameworks (CAISFs) [1]. These frameworks can be produced by using computer-aided design and computer-aided manufacturing (CAD-CAM) technology, a subtractive manufacturing method that requires precisely removing material from a solid block [2–4]. However, the fabrication of intricate geometries has changed with the advent of additive manufacturing (AM) technology, also known as 3-dimensional (3D) printing, resulting in reduced material waste and potentially higher precision [5].

AM technology was defined by the ISO/ASTM 52900 terminology standard as the process of joining materials to make parts from 3D model data [5]. This technology enables the manufacturing of customized frameworks based on anatomical variations, thus enhancing clinical outcomes and precision [6]. Milling hard materials like titanium and its alloys presents challenges as huge material waste, accuracy of framework is determined by the bur size, and high cost. These challenges can be addressed by the use of AM technology including; reduced material waste, design freedom in complex geometries, cost effectiveness and overall optimized production speed [6, 7].

Powder bed fusion (PBF) technology is a category of AM, the most used in dentistry to 3D print metals, it’s categorized into 3 techniques; including selective laser sintering (SLS), selective laser melting (SLM), and electron beam melting (EBM) [8–14]. SLM technique uses a higher power laser type to initially melt and then fuse the external surfaces of the grains [15]. The temperature does not surpass the melting point in the SLS technology, representing a crucial distinction from the SLM and EBM technologies [16, 17].

The clinical success of implant-supported restorations depends on attaining a passive fit between the framework and implant abutments [18]. Passive fit can be described as the lack of strain at the implant-framework interface [19]. A non-passive fit may result in micromovements, screw loosening, fretting damage, bone resorption, and osseointegration disruption [20, 21]. Clinical trials have reported marginal discrepancy values ranging from 150 to 230 µm [19, 22]. Although a clinically acceptable maximum marginal discrepancy value remains unclear, it should not exceed 120 μm [23].

A widely used method for clinical evaluation of framework passive fit is the Sheffield test, also known as the “1-screw test”. The test involves tightening a single screw on the terminal abutment and measuring the marginal discrepancy between the framework and abutments at other implant sites [24]. Ideally, no discrepancy should be present at the unscrewed implant sites. However, a discrepancy below 120 μm is considered clinically acceptable [23]. This method has been employed to evaluate long-span frameworks, whereas the vertical difference is more noticeable and magnified at the other end which implies measuring the discrepancies in the unscrewed implants in proximity and distant to the screwed implant [21, 25]. Recently, the modified 1-screw test has been introduced to ensure adequate framework seating [20]. This includes tightening all the screws, taking them out, leaving one tightened, and measuring the marginal discrepancies in other sites through the use of magnification X-rays or microscopes [26–29].

Most studies rely on microscopic analysis to detect discrepancies at the implant-abutment interface [20]. However, this technique cannot identify mismatches in 3 dimensions or detect distortions occurring in the horizontal plane [25]. Therefore, microcomputed tomography (µCT) has been introduced as a promising alternative to conventional microscopy [2, 4], allowing the X-rays to penetrate the framework, thus capturing the implant-abutment interface and providing a detailed view of 3D marginal discrepancies throughout complete arch restorations [2, 26, 29, 30].

The passive fit of milled titanium frameworks has been reported in many studies [26–29]. However, data on the passive fit of additively manufactured titanium CAISFs are sparse. The purpose of this study was to evaluate the passive fit of milled and additively manufactured titanium CAISFs through 1-screw test by measuring marginal discrepancies for the 3 unscrewed implants at 4 points using µCT. The first null hypothesis was that no difference would be found in the passive fit of milled and additively manufactured titanium CAISFs. The second null hypothesis was that no difference would be found in the marginal discrepancy among implant locations, regardless of proximity to or distant from the screwed implant.

## Methods

The sample size was determined by using a power analysis software program (G*Power version 3.1.9.7) [31]. Based on a previous study [32], a total sample size of 10 (n = 5 per group) was sufficient to achieve a power of 80%. The power analysis considered an effect size (F=0.545), α=0.05, and β=0.2 using Cohen’s test. This sample size was deemed adequate for detecting clinically relevant differences between groups, to examine the null hypothesis that no difference would be found in the passive fit of milled and additively manufactured titanium CAISFs and no difference would be found in the marginal discrepancy among implant locations, regardless of proximity to or distant from the screwed implant.

**Fig. 1:**
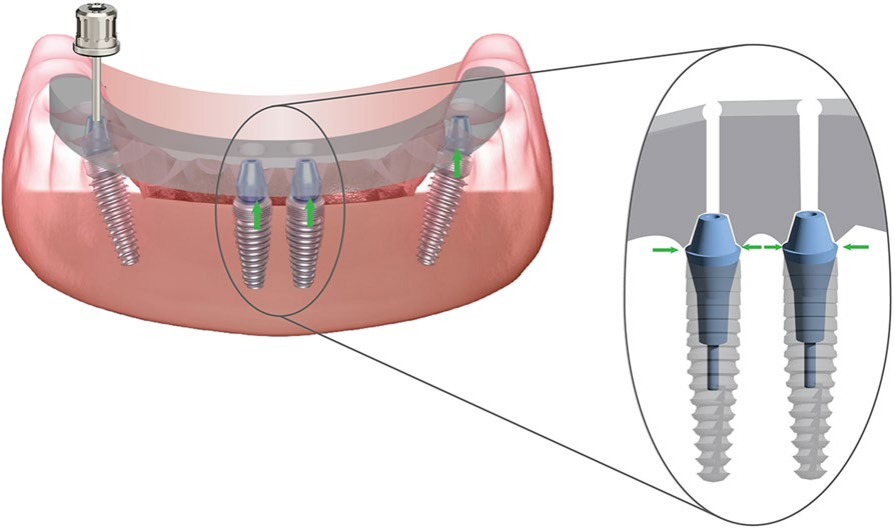
Schematic representation of complete arch implant-supported frameworks configuration and 1-screw test

**Fig.2:**
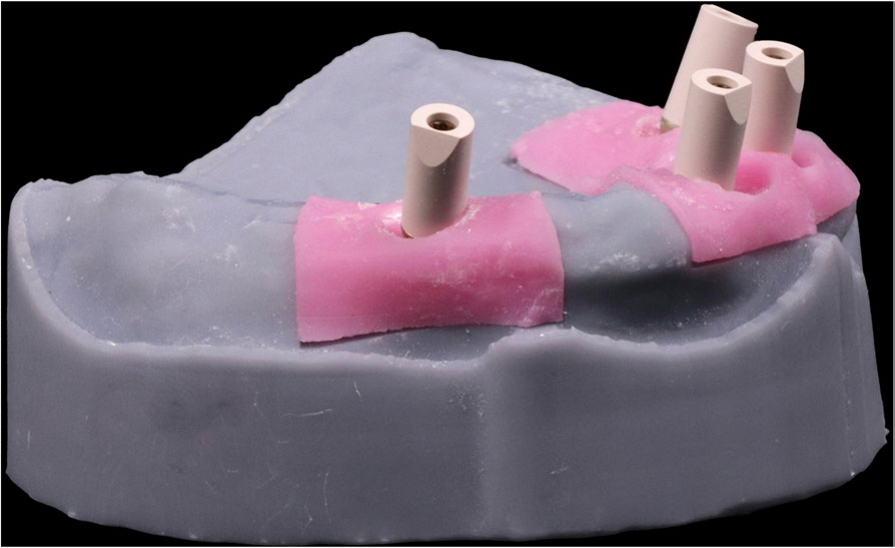
Scan bodies screwed on multiunit abutments for intra oral scanner digitization

Figure 1 displays a schematic of the CAISF configuration and 1-screw testing. An edentulous maxillary model resembling the all-on-4 concept was used, according to a previously published in vitro study [20]. The Model was designed using implant planning CAD software (implant studio, 3shape). Two DICOM files from cone beam computed tomography (CBCT) images (iCAT vision) were imported using a dual scan protocol, the first CBCT showed an edentulous patient wearing a denture under occlusion with radiographic markers on the palate. The second CBCT displayed the denture alone with the same markers. The merging process was automated, and the intaglio surface of the denture was virtually duplicated. Virtual implant placement was carried out following the all-on-four concept. 3D printing technology was used to fabricate the models using LCD technology (Halot Sky-Creality). Two anterior digital analogs were placed parallel to each other in the central incisor region (Repositionable implant analog NC; Institut Straumann AG) and 2 posterior 30-degree angled analogs were placed in the premolar region (Repositionable implant analog NC; Institut Straumann AG). Anterior digital analogs received straight multiunit abutments (NC Screw-retained abutment straight; Institut Straumann AG), while posterior tilted analogs received 30-degree angled multiunit abutments (NC screw-retained abutment, TAN angled 30°; Institut Straumann AG). Scan bodies (CARES Monoscanbody for screw-retained abutment; Institut Straumann AG) were screwed onto multiunit abutments and hand-tightened according to manufacturer instructions (Fig. 2). The model was digitized by using an intraoral scanner (IOS) (Primescan; Dentsply Sirona). Scanner calibration was performed according to manufacturer instructions. A single scan was performed by an experienced operator in a controlled laboratory environment, under standard ambient lightning, and free from dust and moisture. The scanning protocol (posterior-anterior sweep, palatal return path) was followed, in accordance with the manufacturer's recommendations for complete arch scanning. Data were exported as a standard tessellation language (STL) file to a CAD software program (exocad v3.0 Galway; exocad GmbH). A virtual framework was designed to fit directly on the multiunit abutment without an interface. Ten frameworks were produced and allocated to 2 groups according to the manufacturing method (*n=*5): group M, milled from titanium by using CAD-CAM technology and group AM, additively manufactured by using SLM technology.

**Fig. 3:**
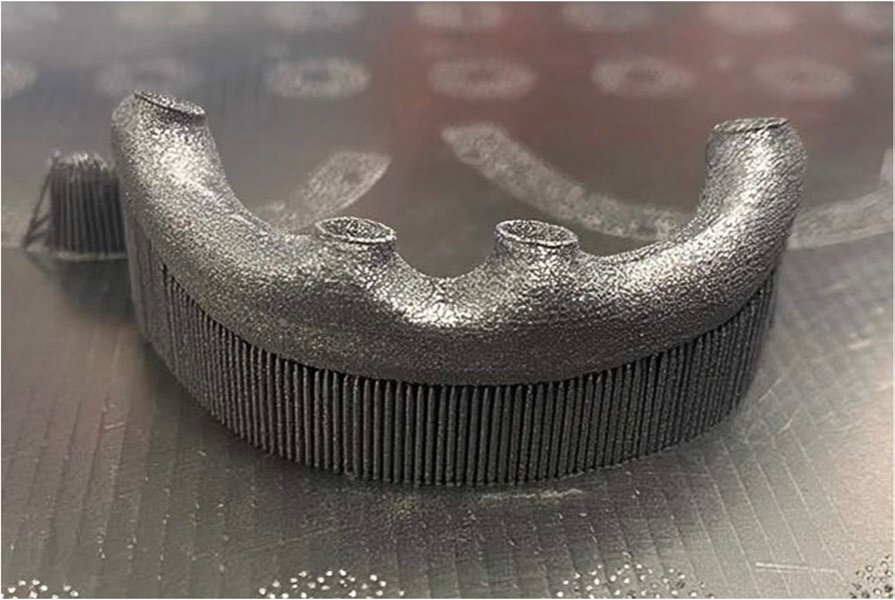
Additively manufactured titanium framework produced by selective laser melting

For group M, the designed framework was sent to a 5-axis milling unit (Coritec 350i pro; imes-icore GmbH), where CAISFs (*n=*5) were milled from a titanium blank alloy (Ti-6AL-4V) (Coritec Titan grade V disc; imes-icore GmbH). For group AM, the initial designed framework was sent to an SLM machine (Ren 500 M; Renishaw), where CAISFs (*n=*5) were additively manufactured from titanium alloy (Ti-6AL-4V) powder (Ti64Gd23; 6 K Additive Inc.) (Fig. 3). The printing machine has a build volume of 286x286x295 mm, an ytterbium fiber laser power of 300 W, a focus diameter of 70 µm, grain sizes ranging from 15 to 45 µm, and layer height 30 µm. In addition, an argon atmosphere has been used to keep oxygen levels below 1300 ppm during printing, thus reducing oxidation and spatter and enhancing overall parts quality. Gas atomized with D10=1 µm, D50=29 µm, and D90=4 µm. Each layer was selectively melted according to the predefined scan path, and the process continued layer upon layer until the final CAISFs were fabricated. The printing process parameters are shown in Table 1.

**Table 1:** Process parameters used for selective laser melting (SLM) of additively manufactured titanium frameworks

**Parameter**	**Value**
Laser power	300 W
Laser speed	1600 mm/s
Hatch distance	80 µm
Layer height	30 µm

**Fig. 4:**
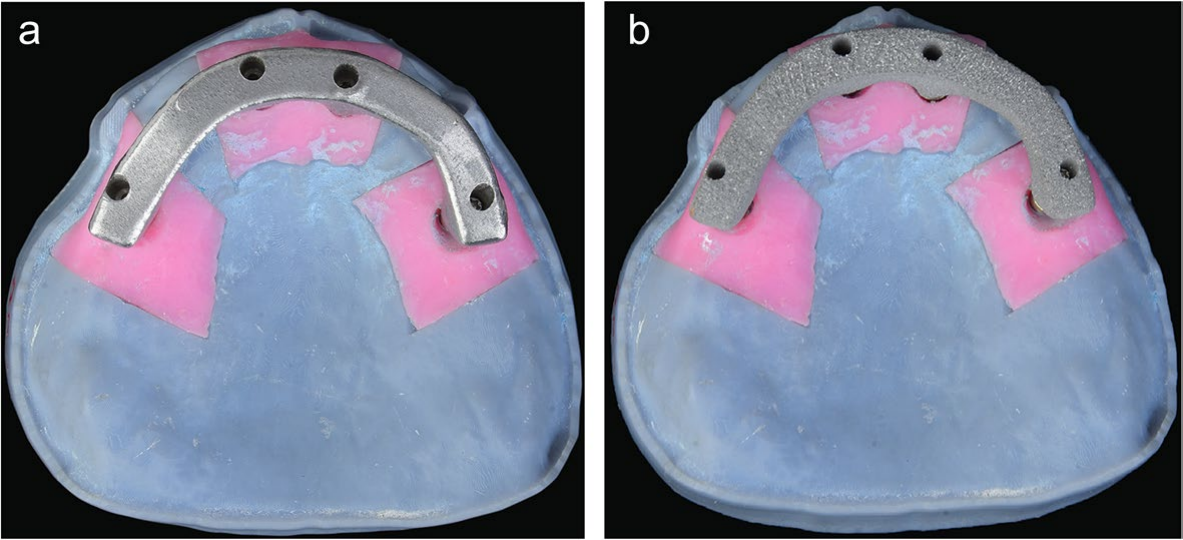
All-on-4 model with complete arch implant-supported frameworks secured on multiunit abutments. **A**, Milled titanium framework. **B**, Additively manufactured titanium framework

All produced frameworks were steam cleaned (EGV 18; Eurocem Srl), seated on multiunit abutments without an interface, and prepared for testing (Fig. 4). Passive fit was assessed with the modified 1-screw test [4, 20]. The Primary outcome to access passive fit involved measuring marginal discrepancies for the 3 unscrewed implants at 4 points using µCT. All framework screws were hand-tightened on the model and then removed except the terminal screw. Subsequently, the retained screw was secured with a torque wrench set at 15 Ncm according to the manufacturer’s instructions. Implant proximity to the screwed abutment was a predefined factor to assess its influence on marginal discrepancy.

**Fig.5:**
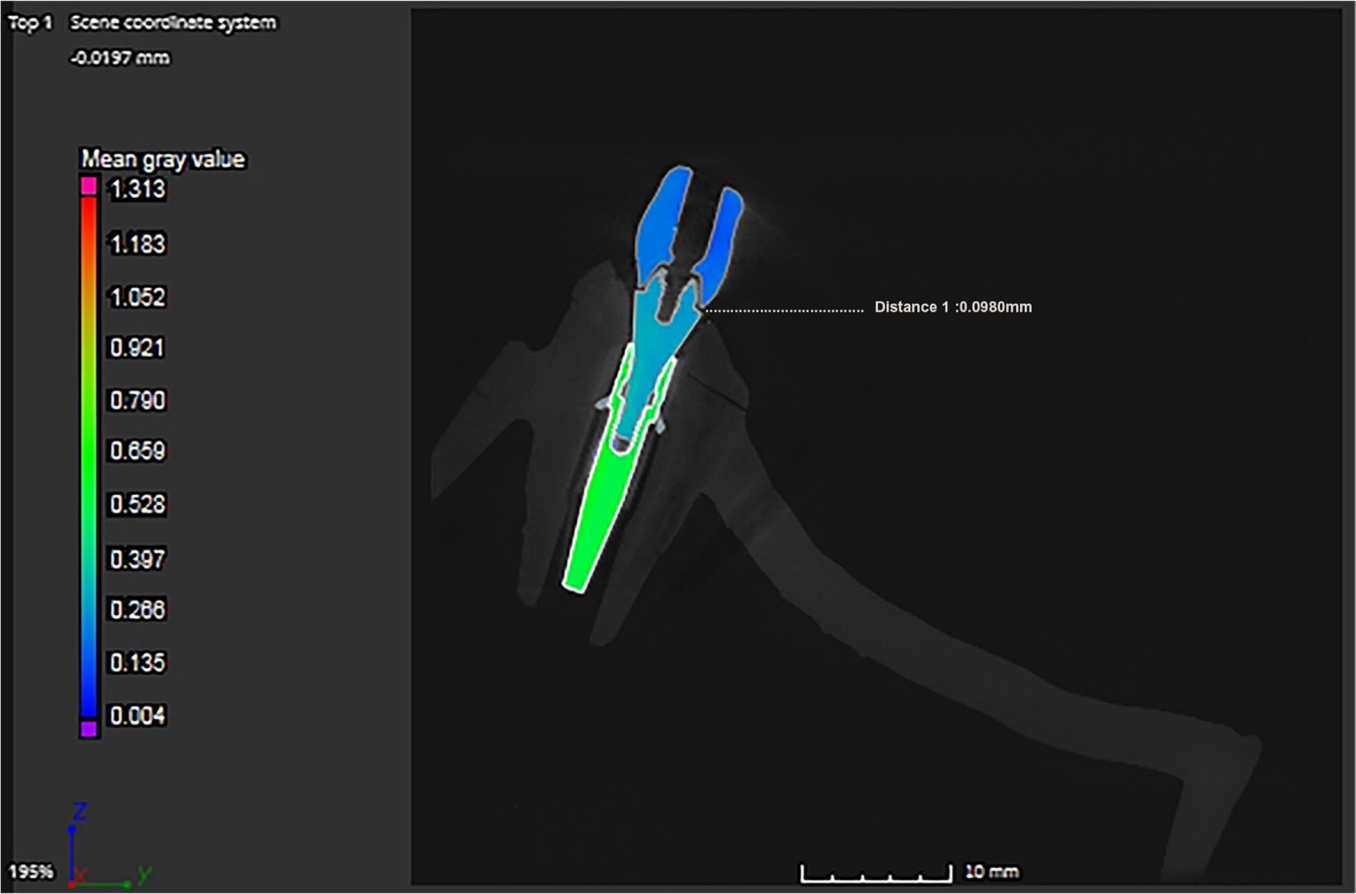
Commercial analysis software program (VGStudio MAX 3.5) for marginal discrepancy analysis of complete arch implant-supported frameworks at abutment-framework interface

**Fig. 6:**
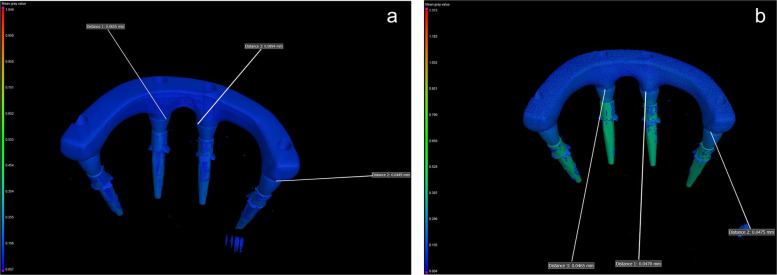
Color difference material X-ray attenuation maps of 3-dimensional (3D) marginal discrepancy values measured at different locations of abutment-framework interface (mesial, buccal, lingual, distal) for each implant. **A**, Milled titanium framework. **B**, Additively manufactured titanium framework.

µCT scanner (Nikon XTH 225 ST; Nikon Metrology Ltd) was used to analyze the marginal discrepancies. The µCT was calibrated with a standard 5-ruby artifact using the manufacturer's protocols. Each titanium CAISF was fixed and scanned in the positioning stage. The scanning parameters are shown in Table 2. All µCT scans were reconstructed in CT pro software program to generate a 3D model, a stage 1 beam hardening artifact correction was used, and no noise filter was applied in the reconstruction process. The data was analyzed using (VGStudio MAX 3.5; Volume Graphics GmbH) (Fig. 5). In the analysis process, the marginal discrepancies on the sagittal axis were measured at 4 predetermined reference points from all aspects (mesial, buccal, lingual, distal) for each implant-abutment interface (Fig. 6). Measurements were carried out with the VG Studio t 3D distance length measurement tool, with values recorded in (µm). All µCT scans were analyzed using a fixed grey value threshold of ISO 50%, ensuring consistency across all specimens. A single experienced operator performed the analysis. All measurements were performed on the same maxillary model and each measurement was repeated 3 times, and the mean value was used for analysis.

**Table 2:** Microcomputed tomography (µCT) scanning configuration parameters

**Parameter**	**Value**
Voxel size	9 µm
Filter	250 µm copper
Exposure	4000 ms
Beam Current	6.8 W
Beam intensity	148 kV

Statistical analysis was performed using Python with StatsModels and Pinguin statistical libraries. The Shapiro-Wilk test of normality and theoretical quantile-quantile (Q-Q) plots were used to assess the normality of the data distribution. The marginal discrepancy values showed non-parametric distribution; therefore Mann-Whitney U test was used to test the effect of the manufacturing method on the marginal discrepancy. Kruskal-Wallis test assessed more than 2 variables, the impact of measuring surfaces (mesial, buccal, lingual, distal) of each implant and implant location on the marginal discrepancy. Post hoc Dunn test for pairwise comparisons was applied when the Kruskal-Wallis test was statistically significant. Aligned rank transform (ART) ANOVA was used to evaluate the effect of the interactions among all factors (α=.05).

## Results

Regarding the passive fit, the mean ±standard deviation of the marginal discrepancy value was 44 ±4 μm for the AM group and 49 ±5 μm for the M group. A statistically significant difference in marginal discrepancy value was found between the 2 manufacturing methods (*P*<.001, F=0.839) (Table 3). Regarding implant location, no significant differences in marginal discrepancy value were found among the 3 implant locations (*P*=.822, F=0.014) (Table 4). Regarding measurement surfaces, during the one-screw test, measurements were taken on 3 implants, with each implant assessed at 4 distinct surfaces.; mesial, buccal, lingual, and distal. Statistically significant differences in marginal discrepancy value were found among the 4 measurement surfaces for each implant-abutment interface (*P*<.001, F=0.704) (Table 5). Moreover, results of the post hoc Dunn test showed that the buccal and lingual surfaces had statistically significantly higher marginal discrepancy mean values than the mesial and distal surfaces (*P*<.001) (Table 5). ART ANOVA showed that the interaction among the effects of the manufacturing method, implant location, and measurement surface was not statistically significant (*P*=.992) (Table 6). Because the voxel size for the µCT analysis was 9 µm, the estimated uncertainty of the dimensional measurements was taken to ±9 µm; thus, the obtained results were presented based on ±standard deviations (SDs).

**Table 3:** Mann-Whitney U test results for comparison between marginal discrepancy values (µm) of manufacturing methods

**Group**				***P***	**Effect size (η**^**2**^**)** **(CI 95%)**
**M**		**AM**			
Median (range)	**Mean ±SD**	**Median (range)**	**Mean ±SD**	<.001*	0.839 (0.466 to 1.212)
51 (42–56)	49 ±5	44 (38–52)	44 ±4		

*AM* Additively manufactured, *M* Milled, *SD* Standard deviation. *Statistically significant at *P*<.05

**Table 4:** Kruskal-Wallis test results for comparison between marginal discrepancy values (µm) of implant locations

**Implant 1**		**Implant 2**		**Implant 3**		***P***	**Effect size (η**^**2**^**)** **(CI 95%)**
Median (range)	**Mean** **±SD**	**Median (range)**	**Mean** **±SD**	**Median (range)**	**Mean** **±SD**	.822	0.014(0 to 0.098)
44 (38–55)	46 ±6	47 (38–56)	47 ±5	46 (40–55)	47 ±5		

*SD* standard deviation

**Table 5:** Kruskal-Wallis test results for comparison between marginal discrepancy values (µm) of measurement surfaces

**Measurement surface**								***P***	**Effect size (η**^**2**^**)** **(CI 95%)**
**Mesial**		**Buccal**		**Lingual**		**Distal**			
Median (range)	**Mean ±SD**	**Median (range)**	**Mean ±SD**	**Median (range)**	**Mean ±SD**	**Median (range)**	**Mean ±SD**	<.001*	0.704 (0.612 to 0.796)
42 (38–48)	41^B^ ±2	53 (44–56)	51^A^ ±3	52 (46–55)	51^A^ ±2	42 (38–49)	42^B^ ±2		

*SD* standard deviation. Different superscript uppercase within same row indicate significant difference (*P*˂.05). *Statistically significant at *P*<.05. Post hoc Dunn test results are indicated by A and B superscripts

**Table 6:** Aligned-rank transform (ART) ANOVA estimating effects of manufacturing method, measurement surface, implant location, and their interactions on marginal discrepancy

**Factor/interaction**	**DF**	**Mean square**	**F**	***P***
Method, A	1	0.148	5.389	<.001*
Surface, B	3	0.091	1.205	.231
Implant, C	2	0.066	0.963	.338
A×B	3	0.133	1.202	.232
A×C	2	0.182	0.960	.339
B×C A×B×C	6 6	0.082 0.0025	0.184 0.00009	.854 .992

*DF* Degree of freedom. *Statistically significant at *P*<.05

## Discussion

This in vitro study evaluated the passive fit of milled and additively manufactured titanium CAISFs by using µCT. The first null hypothesis that no difference would be found in the passive fit of milled and additively manufactured titanium CAISFs was rejected. The mean marginal discrepancy value was 44 ±4 μm for the additively manufactured CAISFs and 49 ±5 μm for the milled ones. These results indicate that the manufacturing method significantly affects the passive fit of the 2 tested groups. However, the second null hypothesis that no difference would be found in the marginal discrepancy among implant locations, regardless of proximity to or distant from the screwed implant was accepted (*P*>.05).

The enhanced passive fit of the additively manufactured CAISFs could be attributed to the AM process that enables precise layer upon layer production of complex geometries without the constraints imposed by classic subtractive technologies such as milling [33]. Studies have shown that AM can alleviate the difficulties associated with hard material machining methods [3, 7]. However, the increased marginal discrepancies of the milled CAISFs could be attributed to the milling bur that can shift towards the center during the machining process, leading to the lateral offset of the inner contour and screw channel, thus resulting in potential inaccuracies [7].This problem is particularly pronounced when fabricating intricate structures for implant prostheses.

The effect of the manufacturing method on the marginal precision of CAISFs has been investigated, with some studies reporting that milling was more accurate than the AM method and others reporting that AM was more accurate for CAISFs [8, 16, 33]. Ciocca et al [33] reported mean marginal discrepancy values ranging from 20 to 35 µm for CAISF titanium frameworks produced by the milling technique and 8 to 22 µm for those made by a hybrid technique involving SLM followed by milling, which was consistent with this study as AM showed less marginal discrepancy values. Our study was consistent with Revilla -León et al [34] where AM titanium frameworks has a 3D marginal discrepancy 60+12.6 µm within clinically accepted range. However, the obtained results were inconsistent with those of Revilla-León et al [8], who reported that milled titanium had lower marginal discrepancies than additively manufactured titanium using EBM technology. It should be noted that EBM is generally accepted to have inferior dimensional accuracy than SLM [10].

Based on the study results, no statistically significant differences could be detected in marginal discrepancy among the 3 implant locations. The obtained results revealed that the position of the implant did not affect the marginal discrepancy, which is consistent with previous studies [1, 4]. In contrast, a previous study reported that the implant proximity to the terminal screwed implant could affect the marginal discrepancy [8].

In the present study, the 3D marginal discrepancies were evaluated by using the modified 1-screw test to ensure proper framework seating [1, 4, 20, 28]. It involves hand tightening all screws, taking them out, leaving one tightened, and measuring the marginal discrepancies on the other implant-abutment interface [4, 20]. μCT was used in the present study to evaluate the marginal discrepancies in milled and additively manufactured titanium frameworks. μCT provides high-resolution, non-destructive 3D imaging, allowing precise examination of internal and external structural details. The μCT scanner settings were optimized for metal analysis to ensure effective penetration of the dense titanium material. Detailed 3D models were reconstructed from the tomographic images, allowing accurate measurements of marginal discrepancies using a modified 1-screw test [2, 4, 30].

The frameworks manufactured in this study were designed to precisely fit onto the multiunit interface (capless technique) without the need for a titanium base (Ti-Base) abutments or a cap to be cemented onto the framework and secured to the multiunit abutment using screws. Unlike restorations supported by natural teeth, intraoral scanning for implant prostheses requires the use of a scan body aligned with the implant shoulder. Precise transfer of coordinates between the implant and prosthetic components is crucial within the CAD software. The alignment of the framework on the multiunit abutments relies on a “library file” provided by the software, since no Ti-Base interface was used, the connection is directly milled, or 3D printed. This file defines the contour and inner structure of the framework, which is essential for the design process [7].

Factors affecting the accuracy of additively manufactured frameworks include the particle size of the metal powder, build orientation, laser power and speed, hatch distance, and system parameters [35, 36]. However, the material layer height and the energy source rate (electron beam or laser) are the most important factors [16]. SLM technology was used in the present study to fabricate titanium frameworks while reducing material waste and production time. This technology melts metal powder by using a ytterbium fiber laser in an argon atmosphere to produce a solid layer 30-μm thick, enabling the fabrication of sturdier titanium frameworks with great precision [35]. In contrast, the EBM technology produces a layer 100-μm thick under normal conditions. Increasing the layer height may accelerate the production but may cause incomplete fusion of the metal powder [16, 32]. This may result in unconsolidated parts, rough surface, reduced mechanical properties, and altered dimensional accuracy, especially when rough surfaces touch the abutments during marginal discrepancy measurements in the modified 1-screw test [14].

Postprocessing of additively manufactured titanium frameworks is crucial for enhancing their surface finish, passive fit, and mechanical properties [37]. However, all produced CAISFs were tested in the as-milled or as-printed condition, to determine the effect of the manufacturing method itself on the passive fit and dimensional accuracy of frameworks, which is crucial for the clinical longevity of CAISFs. Nevertheless, future research is needed to evaluate the effect of postprocessing procedures, including heat treatment and surface finishing, on the passive fit of CAISFs fabricated by AM.

In the present study, the mean marginal discrepancy results for the milled and additively manufactured titanium frameworks ranged from 44 ±4 μm to 49 ±5 μm, thus falling within a clinically acceptable range 120 μm [23]. Although studies on the passive fit of additively manufactured titanium restorations are scarce, the results of this in vitro study showed comparable marginal discrepancy within clinical acceptable range within additively manufactured and milled titanium frameworks. However, data about the long-term performance of additively manufactured titanium restorations are lacking.

Intraoral scanner (Primescan; Dentsply Sirona) was selected to digitize the master model in this study due it’s proven high trueness in digitizing edentulous arches (24 μm −28 μm), supporting its efficacy in clinical applications. To mitigate precision concerns, only a single scan was conducted, considering it a non-significant variable in this context. However, desktop scanners exhibit superior trueness when compared to IOS which might introduce a minor source of variability in digitizing full arches with an IOS which serves as a limitation of this study [20, 38, 39].

Limitations of this study included that the accuracy of AM could change according to different technologies and printing orientations. Further investigations should assess the impact of veneer firing and thermal cycling on the passive fit of frameworks as well as clinical performance. Additionally, the use of IOS to digitize the model may introduce inaccuracies when compared to the superior trueness of desktop scanners; therefore, future studies using desktop scanners are recommended for standardization. The small sample size is another limitation as it may impact statistical robustness, it is recommended that future research involves larger sample sizes to enhance the generalizability of the findings. The findings should be interpreted as exploratory and methodological, rather than directly applicable to clinical decision-making.

## Conclusion

Within the limitations of this in vitro study, the following conclusions were drawn:

1. Additively manufactured titanium CAISFs demonstrated better passive fit than milled ones. However, both techniques produced frameworks with clinical acceptable marginal fit.
2. The proximity of implant location to adjacent screwed implants did not affect marginal discrepancy.

## Data Availability

The datasets used and analyzed during the current study available from the corresponding author on reasonable request.

## References

[1] Yilmaz B, Alshahrani FA, Kale E, Johnston WM. Effect of feldspathic porcelain layering on the marginal fit of zirconia and titanium complete-arch fixed implant-supported frameworks. J Prosthet Dent. 2018;120:71–8. 29426786 10.1016/j.prosdent.2017.11.003

[2] Al-Meraikhi H, Yilmaz B, McGlumphy E, Brantley WA, Johnston WM. Distortion of CAD-CAM-fabricated implant-fixed titanium and zirconia complete dental prosthesis frameworks. J Prosthet Dent. 2018;119:116–23. 28477917 10.1016/j.prosdent.2017.02.003

[3] Katsoulis J, Müller P, Mericske-Stern R, Blatz MB. Cad/cam fabrication accuracy of long- vs. short-span implant-supported fdps. Clin Oral Implants Res. 2015;26:245–9. 25363301 10.1111/clr.12522

[4] Yilmaz B, Kale E, Johnston WM. Marginal discrepancy of CAD-CAM complete-arch fixed implant-supported frameworks. J Prosthet Dent. 2018;120:65–70. 29475755 10.1016/j.prosdent.2017.11.021

[5] ISO/ASTM International. ISO/ASTM 52900:2015: Additive manufacturing — General principles — Terminology. Geneva: ISO; 2015.

[6] Boontherawara P, Chaijareenont P, Angkasith P. Comparing the trueness of 3D printing and conventional casting for the fabrication of removable partial denture metal frameworks for patients with different palatal vault depths: an in vitro study. J Prosthet Dent. 2024;132:434.e1-434.e6. 38845279 10.1016/j.prosdent.2024.05.009

[7] Abou-Ayash S, Schimmel M, Özcan M, Ozcelik B, Brägger U, Yilmaz B. Trueness and marginal fit of implant-supported complete-arch fixed prosthesis frameworks made of high-performance polymers and titanium: an explorative in-vitro study. J Dent. 2021;113:103784. 34419479 10.1016/j.jdent.2021.103784

[8] Revilla-León M, Pérez-López J, Barmak AB, Raigrodski AJ, Rubenstein J, Galluci GO. Implant-abutment discrepancy before and after acrylic resin veneering of complete-arch titanium frameworks manufactured using milling and electron beam melting technologies. J Prosthodont. 2022;31:88–96. 35313021 10.1111/jopr.13422

[9] Velôso DV, Barbin T, Del Rio Silva L, Borges GA, Camacho Presotto AG, Mesquita MF. Additive manufacturing of metallic frameworks supported by the all-on-six implant concept: dimensional precision after veneer layering and spark erosion. Int J Oral Maxillofac Implants. 2022;37:700–8. 35904826 10.11607/jomi.9605

[10] Revilla-León M, Ceballos L, Özcan M. Implant prosthodontic discrepancy of complete-arch Co-Cr implant frameworks manufactured through selective laser melting additive manufacturing technology using a coordinate measuring machine. Int J Oral Maxillofac Implants. 2019;34:698–707. 30892285 10.11607/jomi.6739

[11] Al Jabbari YS, Koutsoukis T, Barmpagadaki X, Zinelis S. Metallurgical and interfacial characterization of PFM Co-Cr dental alloys fabricated via casting, milling or selective laser melting. Dent Mater. 2014;30:e79-88. 24534375 10.1016/j.dental.2014.01.008

[12] Örtorp A, Jönsson D, Mouhsen A, Vult von Steyern P. The fit of cobalt-chromium three-unit fixed dental prostheses fabricated with four different techniques: a comparative in vitro study. Dent Mater. 2011;27:356–63. 10.1016/j.dental.2010.11.015 21163516

[13] Revilla-León M, Sadeghpour M, Özcan M. A review of the applications of additive manufacturing technologies used to fabricate metals in implant dentistry. J Prosthodont. 2020;29:579–93. 32548890 10.1111/jopr.13212

[14] Gokuldoss PK, Kolla S, Eckert J. Additive manufacturing processes: selective laser melting, electron beam melting and binder jetting-selection guidelines. Materials (Basel). 2017;19(10):672. 10.3390/ma10060672 PMC5554053 28773031

[15] Forrester K, Sheridan R, Phoenix RD. Assessing the accuracy of casting and additive manufacturing techniques for fabrication of a complete palatal coverage metal framework. J Prosthodont. 2019;28:811–7. 31115125 10.1111/jopr.13076

[16] Thakur J, Parlani S, Shivakumar S, Jajoo K. Accuracy of marginal fit of an implant-supported framework fabricated by 3D printing versus subtractive manufacturing technique: a systematic review and meta-analysis. J Prosthet Dent. 2023;129:301–9. 34147238 10.1016/j.prosdent.2021.05.010

[17] Svanborg P, Eliasson A, Stenport V. Additively manufactured titanium and cobalt-chromium implant frameworks: fit and effect of ceramic veneering. Int J Oral Maxillofac Implants. 2018;33:590–6. 29763497 10.11607/jomi.6028

[18] Sahin S, Cehreli MC. The significance of passive framework fit in implant prosthodontics: current status. Implant Dent. 2001;10:85–92. 11450418 10.1097/00008505-200104000-00003

[19] Brånemark PI. Osseointegration and its experimental background. J Prosthet Dent. 1983;50:399–410. 6352924 10.1016/s0022-3913(83)80101-2

[20] Eid HS, Zohdy MM, Nour M, Salah T. A comparative analysis of the passivity of fit of complete arch implant-supported frameworks fabricated using different acquisition techniques. J Prosthet Dent. 2024;131:477.e1-477.e8. 38129259 10.1016/j.prosdent.2023.11.032

[21] Jemt T. Failures and complications in 391 consecutively inserted fixed prostheses supported by Brånemark implants in edentulous jaws: a study of treatment from the time of prosthesis placement to the first annual checkup. Int J Oral Maxillofac Implants. 1991;6:270–6. 1813395

[22] Jokstad A, Shokati B. New 3D technologies applied to assess the long-term clinical effects of misfit of the full jaw fixed prosthesis on dental implants. Clin Oral Implants Res. 2015;26:1129–34. 25263818 10.1111/clr.12490

[23] Sachs C, Groesser J, Stadelmann M, Schweiger J, Erdelt K, Beuer F. Full-arch prostheses from translucent zirconia: accuracy of fit. Dent Mater. 2014;30:817–23. 24933230 10.1016/j.dental.2014.05.001

[24] Eisenmann E, Mokabberi A, Walter MH, Freesmeyer WB. Improving the fit of implant-supported superstructures using the spark erosion technique. Int J Oral Maxillofac Implants. 2004;19:810–8. 15623055

[25] Abduo J, Bennani V, Waddell N, Lyons K, Swain M. Assessing the fit of implant fixed prostheses: a critical review. Int J Oral Maxillofac Implants. 2010;25:506–15. 20556249

[26] Kanazawa M, Iwaki M, Minakuchi S, Nomura N. Fabrication of titanium alloy frameworks for complete dentures by selective laser melting. J Prosthet Dent. 2014;112:1441–7. 25258261 10.1016/j.prosdent.2014.06.017

[27] Revilla-León M, Meyer MJ, Özcan M. Metal additive manufacturing technologies: literature review of current status and prosthodontic applications. Int J Comput Dent. 2019;22:55–67. 30848255

[28] Yilmaz B, Guo X, Schimmel M, Abou-Ayash S. Effect of industrial scanner and framework material interaction on the marginal gaps of CAD-CAM complete arch implant frameworks. J Prosthet Dent. 2023;130:723–30. 34998580 10.1016/j.prosdent.2021.10.013

[29] Yunizar MF, Watanabe M, Ichikawa T. Current development status of additive manufacturing technologies for fabricating removable partial denture frameworks: a literature review. Int J Comput Dent. 2022;25:5770. 35322653

[30] Attia MA, Blunt L, Bills P, Tawfik A, Radawn M. Micro-CT analysis of marginal and internal fit of milled and pressed polyetheretherketone single crowns. J Prosthet Dent. 2023;129:906.e1-906.e10. 37072286 10.1016/j.prosdent.2023.03.018

[31] Faul F, Erdfelder E, Lang AG, Buchner A. G*power 3: a flexible statistical power analysis program for the social, behavioral, and biomedical sciences. Behav Res Methods. 2007;39:175–91. 17695343 10.3758/bf03193146

[32] Barbin T, Velôso DV, Del Rio SL, Borges GA, Presotto AGC, Barão VAR, et al. 3D metal printing in dentistry: an in vitro biomechanical comparative study of two additive manufacturing technologies for full-arch implant-supported prostheses. J Mech Behav Biomed Mater. 2020;108:103821. 32469723 10.1016/j.jmbbm.2020.103821

[33] Ciocca L, Meneghello R, Savio G, Scheda L, Monaco C, Gatto MR, et al. Manufacturing of metal frameworks for full-arch dental restoration on implants: a comparison between milling and a novel hybrid technology. J Prosthodont. 2019;28:556–63. 31038248 10.1111/jopr.13067

[34] Revilla-León M, Ceballos L, Martínez-Klemm I, Özcan M. Discrepancy of complete-arch titanium frameworks manufactured using selective laser melting and electron beam melting additive manufacturing technologies. J Prosthet Dent. 2018;120:942–7. 30006219 10.1016/j.prosdent.2018.02.010

[35] Barbin T, Borges GA, Jardini AL, Mesquita MF. Hot isostatic pressing as an alternative thermo-mechanical treatment for metallic full-arch implant-supported frameworks obtained by additive and subtractive manufacturing technology: Vertical and horizontal fit, screw removal torque, and stress analysis. J Prosthodont. 2024;33(S1):70–80. 38513224 10.1111/jopr.13842

[36] Hwang S, An S, Robles U, Rumpf RC. Process parameter optimization for removable partial denture frameworks manufactured by selective laser melting. J Prosthet Dent. 2023;129:191–8. 34119322 10.1016/j.prosdent.2021.04.021

[37] Peng PW, Hsu CY, Huang HY, Chao JC, Lee WF. Trueness of removable partial denture frameworks additively manufactured with selective laser melting. J Prosthet Dent. 2022;127:122–7. 33223197 10.1016/j.prosdent.2020.06.035

[38] Denneulin T, Rignon-Bret C, Ravalec G, Tapie L, Bouter D, Wulfman C. Accuracy of complete-arch implant digital scans: effect of scanning protocol, number of implants, and scan body splinting. Int J Prosthodont. 2023;36(2):219–27. 36288490 10.11607/ijp.7332

[39] Revell G, Simon B, Mennito A, Evans ZP, Renne W, Ludlow M, et al. Evaluation of complete-arch implant scanning with 5 different intraoral scanners in terms of trueness and operator experience. J Prosthet Dent. 2022;128(4):632–8. 33832761 10.1016/j.prosdent.2021.01.013

